# Histological evaluation of the aortic wall response following endovascular aneurysm repair and endovascular aneurysm sealing

**DOI:** 10.1016/j.jvssci.2023.100101

**Published:** 2023-03-23

**Authors:** Laura E. Bruijn, Jan M.M. Heyligers, Patrick W. Vriens, Jacoba van Rhijn, Joy Roy, Jaap F. Hamming, Gabor Gäbel, Jan H.N. Lindeman

**Affiliations:** a Division of Vascular Surgery, Department of Surgery, Leiden University Medical Center (LUMC), Leiden, the Netherlands; b Department of Surgery, Elisabeth-TweeSteden Ziekenhuis, Tilburg, the Netherlands; c Department of Medical & Clinical Psychology, Tilburg University, Tilburg, the Netherlands; d Department of Vascular Surgery, Karolinska University Hospital Stockholm, Stockholm, Sweden; e Department of Vascular Surgery, Helios Klinikum Krefeld, Krefeld, Germany

**Keywords:** Abdominal aortic aneurysm, Endoleak, Endovascular aneurysm repair, Vascular remodeling, Histology

## Abstract

**Objective:**

The Nellix endovascular aneurysm sealing (EVAS) system was developed as an alternative to conventional endovascular aneurysm repair (EVAR) to minimize endoleaks. A significantly higher failure rate of EVAS may be related to an interaction between the filled endobags and the AAA wall. In general, biological information on aortic remodeling after traditional EVAR is scarce. In this light, we provide here the first histologic evaluation of aneurysm wall morphology after EVAR and EVAS.

**Methods:**

Fourteen histological human wall samples of EVAS and EVAR explantation were systematically analysed. Primary open aorta repair samples were included as reference.

**Results:**

Compared with primary open aortic repair samples, endovascular repair aortic samples were characterized by more pronounced fibrosis, a greater number of ganglionic structures, decreased cellular inflammation, less calcification, and a lower atherosclerotic load. EVAS was specifically associated with the presence of unstructured elastin deposits.

**Conclusions:**

The biological response of the aortic wall after endovascular repair resembles the maturation process of a scar rather than a bona fide healing response. Moreover, the inflammatory response in the aortic wall after placement of endovascular protheses is less prominent than after primary open repair. A specific post-EVAS aortic wall characteristic was unstructured elastin fragments.

Endovascular aneurysm repair (EVAR) established itself as the gold standard for elective abdominal aortic aneurysm (AAA) repair. The Achilles heel of EVAR is the endoleak and consequent need for postrepair surveillance and reinterventions. In an attempt to minimize incident endoleaks, the Nellix endovascular aneurysm sealing (EVAS) system (Endologix Inc, Irvine, CA)^1^ was introduced. This system is based on an endobag-lined, balloon-expandable stent. After positioning of the endograft, two endobags are filled with a polymer under active pressure control to actively seal the aneurysm sac.

Unexpectedly, the Nellix System^2^ was associated with a significantly higher rate of failures than conventional EVAR systems,^3^ in particular, type Ia endoleaks, graft migration, and secondary ruptures. These observations are remarkable and may reflect an interaction between the endobags and the AAA wall. Because devices analogous to the Nellix system are being developed to overcome endoleaks, this aspect is of potential clinical relevance. In general, biological information of aortic remodeling after traditional EVAR is scarce.

To address this deficit, we performed a first histological evaluation to inventory histological changes after endovascular repair, in particular EVAS.

## Methods

This exploratory study includes wall samples from 10 patients who required explantation of the Nellix system because of graft failure (endoleak, device migration, or sac enlargement). Time to explantation ranged from 12 to 62 months. Further clinical characteristics are provided in Table I.

**Table I:** Patient characteristics

Case number (EVAS/EVAR)	Sex	Age, years	Time of prothesis in situ, months	Reason explantation	Smoking	Statin use	Antihypertension medication use	Diabetes	Genetic form of AAA	Cerebrovascular accident	History of coronary atherosclerosis^a^	Atrial fibrillation	Peripheral arterial disease^b^	Chronic obstructive pulmonary disease^c^	Inflammatory disease
1-EVAS	F	80	26	Endoleak type II^d^	Former	No	Yes	No	No	Yes	Yes	No	Unknown	Unknown	No
2-EVAS	M	80	13	Endoleak type Ia^e^ and sac enlargement	No	Yes	Yes	No	No	No	No	Yes	No	Unknown	No
3-EVAS	F	68	29	Endoleak type Ia^e^ and device migration^f^	Former	Yes	Yes	Yes	No	No	No	No	Yes	Yes	No
4-EVAS	M	73	24	Device migration^f^	Yes	Yes	Yes	No	No	No	No	No	Unknown	No	No
5-EVAS	M	75	23	Sac enlargement	Yes	Yes	Yes	No	No	No	No	No	Unknown	No	No
6-EVAS	F	64	12	Flinching endobags	Yes	Yes	Yes	No	No	No	No	Yes	Unknown	Unknown	No
7-EVAS	M	77	62	Sac enlargement and device migration^f^	No	Yes	Yes	No	No	No	No	No	Yes	Unknown	No
8-EVAS	F	74	33	Device migration^e^	Former	Yes	Yes	No	No	No	No	No	Unknown	Yes	Grave's disease
9-EVAS	M	68	42	Endoleak type Ia^e^ and sac enlargement	Former	Yes	Yes	No	No	Yes	Yes	No	Unknown	Yes	No
10-EVAS	M	66	32	Endoleak type Ia,^e^ sac enlargement and device migration^f^	Former	Yes	Yes	No	No	No	Yes	No	Yes	Yes	Hashimoto's disease
11-EVAR	M	78	100	Atherosclerotic occlusion of iliac vessels	Former	No	No	No	No	No	No	No	Yes	Yes	No
12-EVAR	M	79	92	Graft infection with regressed sac	Former	No	Yes	No	No	No	No	No	No	No	No
13-EVAR	M	75	20	Graft infection	Former	Yes	No	No	No						
14-EVAR	M	72	61	Type II and III endoleak	Former	Yes	Yes	No	No						

*F*, Female; *M*, male.

a History of myocardial infarction or angina pectoris.

b Intermittent claudication, an abnormal ankle-brachial index (<0.9) or interventions such as Percutaneous Transluminal Angioplasty.

c Both clinical symptoms and abnormal pulmonary function testing.

d Type II endoleak: endoleak due to flow through open collateral arteries.

e Type Ia endoleak: persistent proximal inflow due to incomplete sealing.

f Device migration defined as any stent graft movement of ≥4 mm from the predefined reference.

EVAR reference samples were obtained from four patients who required an aortic bifurcation prosthesis for respectively endoleaks, an atherosclerotic iliac occlusion following earlier successful EVAR, reoperation after endoleak or (suspected) graft infection. Aortic reference samples were obtained during elective open repair. Details of the open repair samples included in this study were described elsewhere.^4^

From each patient, one tissue block was randomly chosen for this study. Sequential (4-μm) sections were stained with Hematoxylin and Eosin and the Movat Pentachrome staining, and were independently graded by two observers (L.B., J.L.) using the histological consensus classification scheme for AAA.^4^

Immunohistochemistry was performed to visualize subtypes of leukocytes. Details of the primary antibodies are provided in Table II. Heat-induced (TRIS/EDTA pH 9.2) antigen retrieval was performed. All primary antibodies were diluted in 1% bovine serum albumin/phosphate-buffered saline and were incubated overnight at 4°C. Serum-free protein block (Dako, Glostrup, Denmark) was performed for 10 minutes before incubation if necessary. Endogenous peroxidase activity was blocked with a 20-minute incubation with 0.3% hydrogen peroxide. The Envision/DAB (Dako) system was used for visualization. Nuclei were counter stained by Mayer's hematoxylin (Merck Millipore, Amsterdam, the Netherlands). All stainings for a given antibody were processed in a single batch. Images were captured by means of a digital microscope (Philips IntelliSite Pathology Solution Ultra-Fast Scanner, Philips Eindhoven, the Netherlands).

**Table II:** Primary antibodies used in the present study

Antibody, clone	Target	Host isotype, subclass	Protein block	Dilution	Source
CD4, SC7219	T helper cells	Polyclonal rabbit	Yes	1:800	Santa Cruz Biotechnology
CD8, C8/144B	Cytotoxic T cells and suppressor T cells	Monoclonal mouse, IgG1	No	1:200	Dako
CD68, KP1	Macrophages and dendritic cells	Monoclonal mouse, IgG1	No	1:6000	Dako
CD20, L26	Mature B cells and follicular dendritic cells	Monoclonal mouse IgG2a	No	1:4000	Dako
MPO, A0398	Neutrophils	Polyclonal rabbit	No	1:6000	Dako
Tryptase, AA1 (M7052)	Mast cells	Mouse IgG1	No	1:5000	Dako
Vimentin, 3B4	Mesenchymal cells^5^	Mouse IgG2a	No	1:2000	Dako
Tropomyosin, sc-74480	Contractile cells^5^	Mouse IgG2b	No	1:8000	Santa Cruz Biotechnology
α-Smooth muscle actin, 1A4	Contractile cells^5^	Mouse IgG2a	No	1:4000	Dako

## Results

The clinical characteristics of the selected EVAS and EVAR cases are described in Table I. The median age was 75 years (interquartile range [IQR], 68-78 years), and 67% of the patients were male. The majority of the patients were treated with statins (75%) and/or antihypertensives (92%). The median wall thickness of the EVAS wall samples was 1.3 mm (IQR, 0.72-1.58 mm) as compared with 2.45 mm and 1.78 mm for the respective EVAR samples and 1.01 mm (IQR, 0.74-1.32 mm) for the reference (elective open repair) AAA wall samples (n = 44).

**Fig 1:**
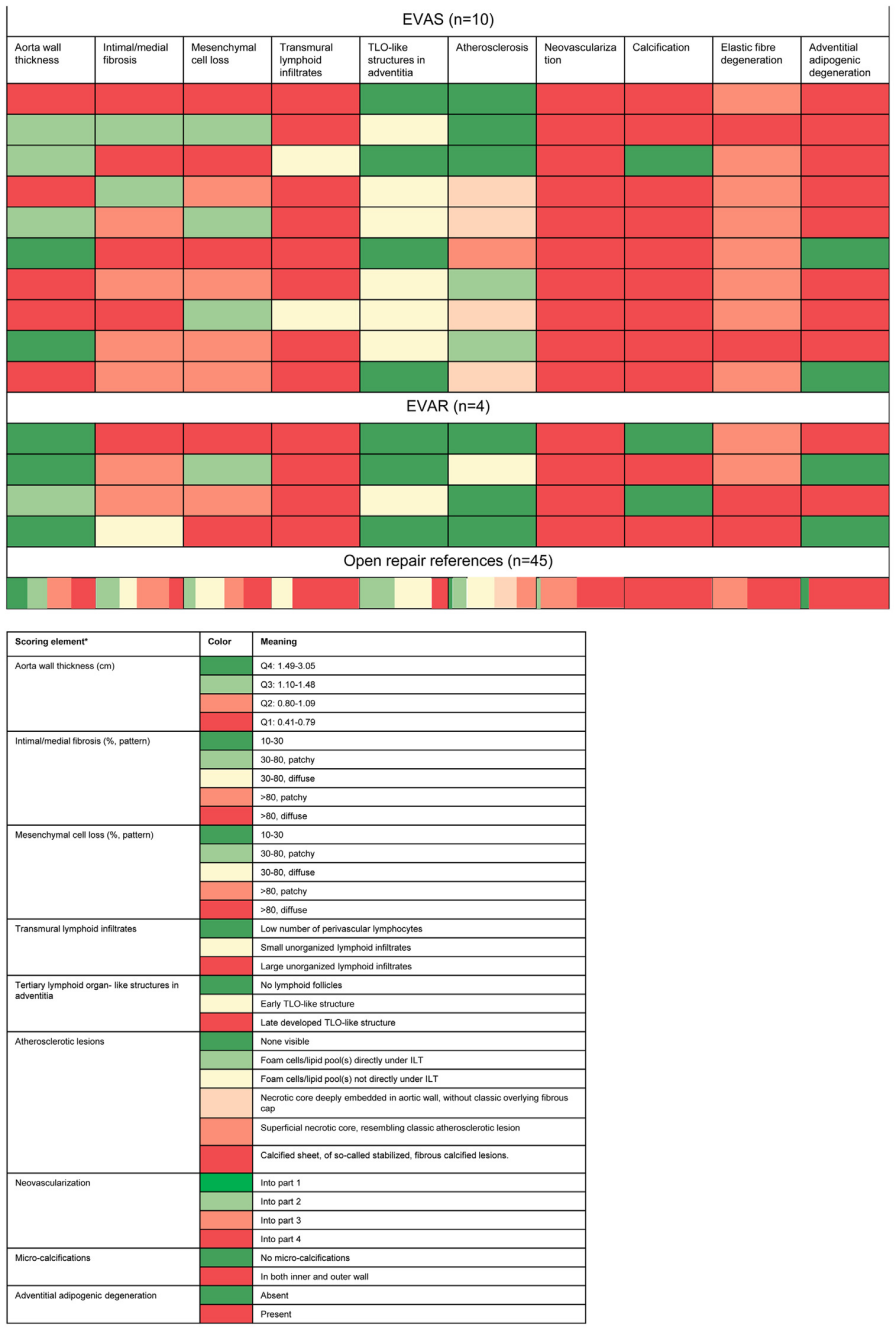
Characteristics of the aortic wall after endovascular aneurysm sealing (*EVAS*) and endovascular aneurysm repair (*EVAR*) compared with open repair, visualized in a heatmap.

**Fig 2:**
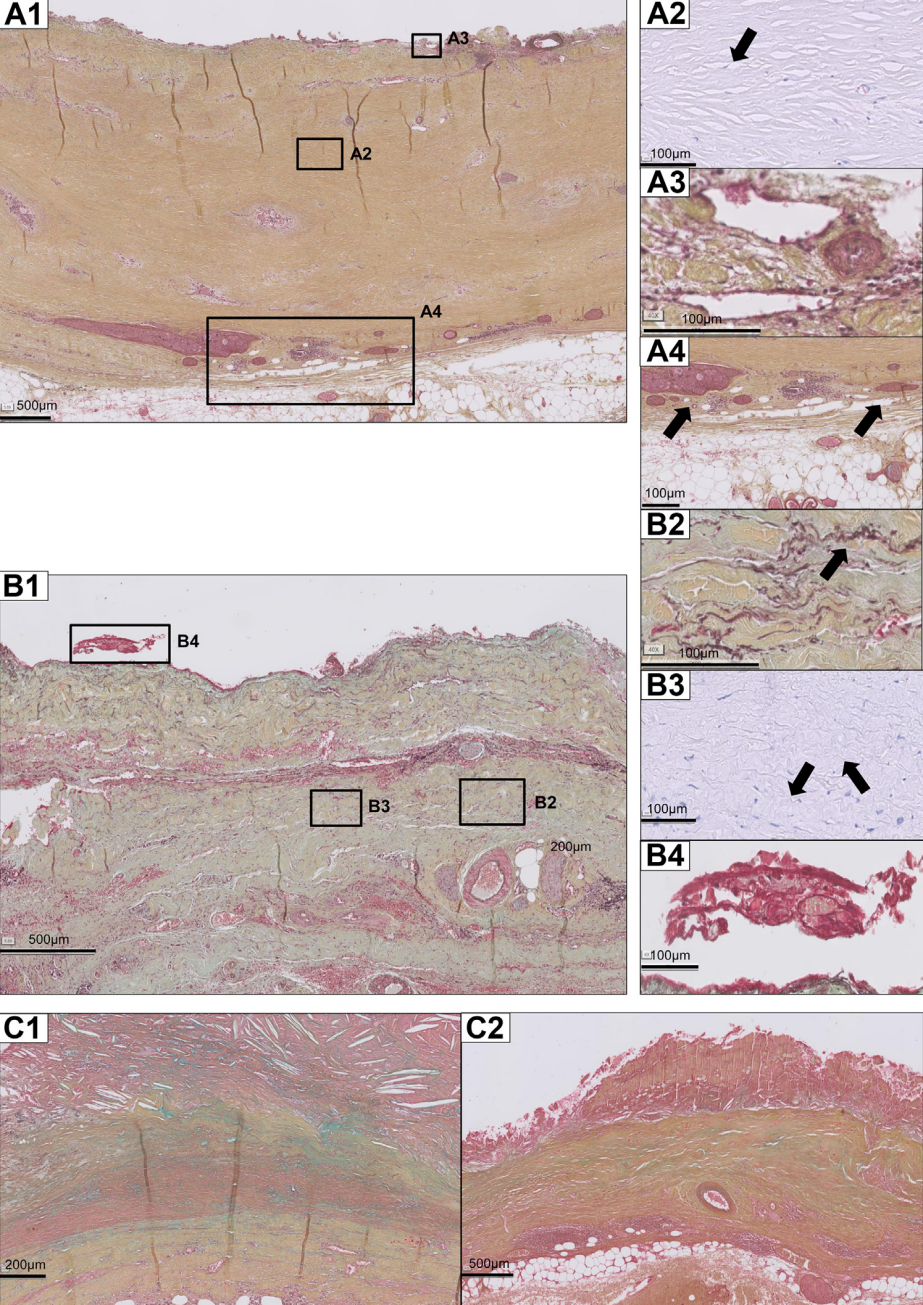
Histological characteristics of the aortic wall after endovascular aneurysm sealing (EVAS) and endovascular aneurysm repair (EVAR) compared with open repair. Movat Pentachrome staining of post-EVAR (**A1**) and post-EVAS (**B2**) aortic wall samples, compared to open repair (**C1/C2**) samples. Note that open repair samples are characterized by a substantial variability in cellular composition^4^; **C1** represents a mesenchymal cell-rich wall sample, while **C2** illustrates a sample that is mesenchymal cell-depleted, but that contains extensive leukocyte infiltrations. Aneurysm walls are primarily composed of mesenchymal cells (predominantly smooth muscle cells; elongated cells in *red* in Movat. Nuclei stain *purple*). Extracellular matrix aspects highlighted in the Movat stain are elastin (*black*), collagen (*yellow*), proteoglycans (*blue*); *green* represents colocalization of proteoglycans and collagen. Both all AAA samples were characterized by extensive elastin loss and fibrosis: the extracellular matrix is rich in collagen (ochre in the Movat staining **A1**/**B1**), while poor in mesenchymal cells (**A2/B3**:corresponding H&E staining; *arrow points* to a nucleus of a mesenchymal cell. Neovessel formation is observed up into the intima (**A3**). Appearance of ganglion-like structures in the adventitial layer is a notable features of the post EVAR and EVAS samples (**A4**, *arrows* highlight two examples). Remnants of intraluminal thrombi were observed in EVAS wall samples (**B4**) and open repair samples, but were absent in the post-EVAR samples. Differences in biological responses are observed for the EVAR and EVAS repair: areas of unstructured elastin deposition were found in EVAS (**B2**, *arrow*); while in conventional EVAR and the reference open repair samples are devoid of elastin.

**Fig 3:**
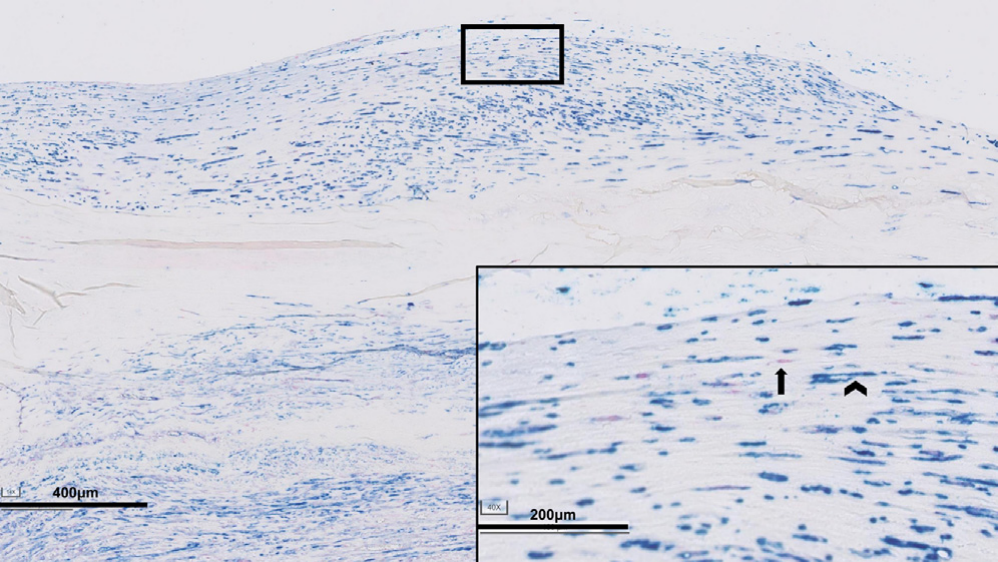
Mesenchymal cell density in post endovascular repair samples. Smooth muscle cells, myofibroblasts and fibroblasts in a post EVAS samples. Mesenchymal cell (elongated cells) are identified by Vimentin staining (*red*). The mesenchymal cell population is dominated by smooth muscle cells and myofibroblastic cells (identified by αSMA and Tropomyosin staining [*blue*]).^5^ Few single Vimentin+ elongated cells fibroblasts are present (*arrow*).

**Fig 4:**
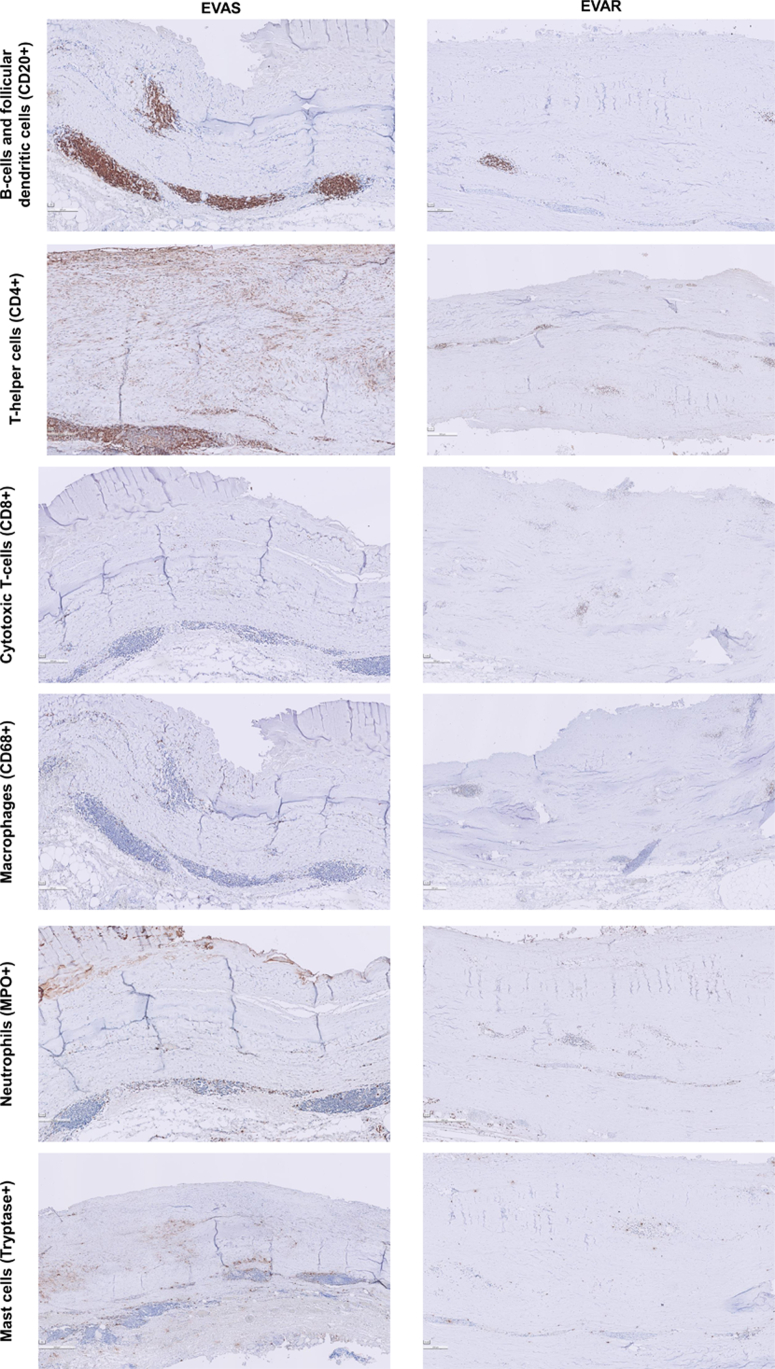
Inflammatory cells present in endovascular repair samples. *EVAR*, endovascular aneurysm repair; *EVAS*, endovascular aneurysm sealing.

Exploration of histological features (consensus classification scheme for AAA)^4^ in the EVAS samples, EVAR samples, and open repair reference samples are summarized in a heatmap (Fig 1). Post-EVAR and post-EVAS samples showed profound fibrosis and depletion of the vascular mesenchymal cell population ([myo]fibroblastic cells and smooth muscle cells)^6^ (Fig 2, *A1-A2*, *B1-B3*, and *C1-C2*). Mesenchymal cell identity was confirmed by vimentin staining, and contractile cells (myofibroblastic cells and smooth muscle cells) by tropomyosin and α-Smooth muscle actin staining (Fig 3). Post-EVAS samples associated with a low amount of adventitial tertiary follicles and fewer transmural leucocytic aggregates compared with open repair samples. B cells were located primarily in these follicles (Fig 4), whereas T cells were mainly present in diffuse infiltrates throughout the aortic wall. T helper cells were the main contributor to immune cell infiltration in postendovascular repair samples.

The EVAR samples with suspected graft infection showed more cellular inflammation than the noninflamed EVAR samples. However, neutrophilic infiltration in this specimen was limited (Fig 4). Other aspects of the adaptive immune cell signature are also illustrated in Fig 4; macrophages and mast cells were present diffusely in small amounts throughout all the vessel wall layers. Remnants of intraluminal thrombi were observed in EVAS wall samples and reference samples (Fig 2, *B4*), but could not be identified in the post-EVAR samples. In some post-EVAS specimens, unstructured elastin fragments were identified, whereas (almost) complete elastolysis was found in post-EVAR and post open repair samples (Fig 2, *B2*).

A universal characteristic of both EVAR and EVAS was extension of neovessels up into the inner intima (Fig 2, *A3*), a feature observed rarely in the reference AAA samples, as well as a more prominent presence of ganglion-like structures in the adventitial layer (Fig 2, *A4*). Calcifications and atherosclerotic lesions appear less prominent in the EVAR and EVAS samples. The extend of adventitial adipogenic degeneration^7^ was similar in the EVAR, EVAS, and reference samples.

## Discussion

Features of aortic wall remodeling after endovascular repair have been linked to clinical outcomes (ie, sac regression is considered to represent treatment success^8^). Remarkably, extremely limited biological data regarding post endovascular repair remodeling are available, and the unanticipated high late/secondary failure rate of the Nellix EVAS system remains an enigma. Because it cannot be ruled out that the high failure rate of the Nellix system relates to a biological interaction between the system and the aneurysm wall, we considered this exploratory evaluation relevant. This histological evaluation of post-EVAS and post-EVAR vascular remodeling implies an absent bona fide healing response (ie, scarring rather than reinstatement of normal vascular structures), a decreased (cellular) inflammatory response, and maturation of the fibrotic response (hypertrophic scarring^9^). Scarring and quenching of the cellular inflammatory response occurred despite an aggravated angiogenic response, which potentially indicated a hypoxic response after endovascular repair.

Areas of unstructured elastin deposition were found in the post-EVAS specimens and could be a reflection of a biological interaction between EVAS and the aneurysm wall. Although endobag rupture and gross exposure of the polymer to the aortic wall were not found in these patients, it cannot be ruled out that polymer-osis^10^ after deployment of the Nellix system triggered a mild biological responses that resulted in (unstructured) elastin deposition. Endoleaks could also not be excluded as a source for aberrant elastin deposition. In four of the EVAS patients, an endoleak type Ia was detected, but in two of these cases complete elastolysis was found. We consider it unlikely that the observed differences between EVAS and EVAR relate to a difference in time to explantation, considering the uniform histological pattern within the EVAS group, and the variances in time to explantation.

The common pattern of a decreased inflammatory and enhanced fibrotic response in EVAS and EVAR compared with open repair may relate to a decrease in wall and/or shear stress, as well as fading of the adhering luminal thrombus, and is in line with the hypothesis that (aspects of) the prominent cellular immune responses in AAA are secondary.^11^ Resolution of the thrombus may also account for the reduced atherosclerotic burden (decreased cholesterol burden from erythrocytes that would be trapped in the adhering thrombus^12^). A nonexclusive explanation for decreased atherosclerotic burden is a shielding effect of the EVAR/EVAS, where the endoprothesis shields the aortic wall from lipoprotein exposure from the luminal blood flow and the aortic endothelium.

This exploratory study provides a unique first biological insight into post-EVAR and EVAS wall remodeling. However, our study has several limitations. First, post-EVAR explantation, in particular after successful deployment, is rare, and we were only able to include four samples. Therefore, all findings should be considered in the context of the observational and exploratory nature of this study; as a consequence, mechanistic insight is limited. Although possible sex difference(s) in biological responses is of obvious interest, the limited number of samples from female patients unfortunately did not allow for separate analysis. Information obtained was from the (former) aneurysm sac; aortic neck samples were not available. Finally, it cannot be excluded that information relating to the thrombus is interfered by stent manipulation during explantation.
